# Who attends antenatal care and expanded programme on immunization services in Chad, Mali and Niger? the implications for insecticide-treated net delivery

**DOI:** 10.1186/1475-2875-10-341

**Published:** 2011-11-13

**Authors:** Meredith Carlson, Lucy Smith Paintain, Jane Bruce, Jayne Webster, Jo Lines

**Affiliations:** 1 Department of Disease Control, London School of Hygiene and Tropical Medicine, Keppel Street, London WC1E 7HT, UK

## Abstract

**Background:**

Malaria remains one of the largest public health problems facing the developing world. Insecticide-treated nets (ITNs) are an effective intervention against malaria. ITN delivery through routine health services, such as antenatal care (ANC) and childhood vaccination (EPI), is a promising channel of delivery to reach individuals with the highest risk (pregnant women and children under five years old). Decisions on whether to deliver ITNs through both channels depends upon the reach of each of these systems, whether these are independent and the effectiveness and cost effectiveness of each. Predictors of women attending ANC and EPI separately have been studied, but the predictors of those who attend neither service have not been identified.

**Methods:**

Data from Chad, Mali and Niger demographic and health surveys (DHS) were analyzed to determine risk factors for attending neither service. A conceptual framework for preventative health care-seeking behaviour was created to illustrate the hierarchical relationships between the potential risk factors. The independence of attending both ANC and EPI was investigated. A multivariate model of predictors for non-attendance was developed using logistic regression.

**Results:**

ANC and EPI attendance were found to be strongly associated in all three countries. However, 47% of mothers in Chad, 12% in Mali and 36% in Niger did not attend either ANC or EPI. Region, mother's education and partner's education were predictors of non-attendance in all three countries. Wealth index, ethnicity, and occupation were associated with non-attendance in Mali and Niger. Other predictors included religion, healthcare autonomy, household size and number of children under five.

**Conclusions:**

Attendance of ANC and EPI are not independent and therefore the majority of pregnant women in these countries will have the opportunity to receive ITNs through both services. Although attendance at ANC and EPI are not independent, delivery through both systems may still add incrementally to delivery through one alone. Therefore, there is potential to increase the proportion of women and children receiving ITNs by delivering through both of these channels. However, modelling is required to determine the level of attendance and incremental potential at which it's cost effective to deliver through both services.

## Background

An estimated 3.4 billion people globally are at risk of malaria with approximately 243 million cases and one million deaths annually [1]. Eighty-five percent of malaria cases and 90% of malaria deaths occur in Africa, with pregnant women and children under five years being especially at risk [1]. The sixth United Nations Millennium Development Goal aims to fight malaria, with a target of halving the incidence by 2015. One measure of success towards this goal is the proportion of children under five and proportion of pregnant women who sleep under an insecticide-treated mosquito net (ITN) [2].

ITNs are a simple, effective and cost-effective malaria intervention that can reduce incidence of clinical malaria by at least 50% and save 5.5 lives for every 1000 children under five years old protected with an ITN per year [3]; use of an ITN during pregnancy reduces low birth weight, maternal anaemia and risk of miscarriage [4]. Delivery of ITNs is complex; there are numerous delivery sectors (public, mixed public-private and private), delivery channels (routine services and campaigns) and subsidy categories (free, partially subsidized and unsubsidized). The World Health Organization (WHO) recommends a combination strategy of "catch up" and "keep up" activities to achieve and maintain sustainable universal ITN ownership and use [5].

Delivery through mass campaigns has been shown to be effective in rapidly reaching a large proportion of the target population, thus "catching up" the population in terms of ITN coverage. However, these nation-wide universal coverage campaigns are only efficient if conducted every three to four years, aiming to cover every sleeping space during the effective period of a long-lasting insecticide treated net's (LLIN) lifespan. For example, integrating delivery with a measles vaccination campaign in Zambia increased household coverage from 21% to 88% in rural areas and from 49% to 82% in urban areas [6]. Integration of ITN delivery with a polio vaccine campaign in Niger resulted in increased household ownership from 6% to 65% [7].

Delivery through routine services is intended to be a complement to campaign delivery, not a substitute for it; the aim is to "keep up" ITN coverage with a continuous flow of nets to pregnant women and children born since the last mass campaign [8]. Routine ITN delivery channels include antenatal care (ANC) clinics, the expanded programme on immunization (EPI) and other maternal and child health (MCH) clinics [9]. Eritrea has adopted a national malaria control programme that includes ITN distribution strictly through ANC and community health agents, which has resulted in 62% household ownership [10]. Furthermore, 100% household coverage of LLINs delivered at the first ANC meeting was maintained after five to seven months in the Adjumani district of Uganda [11]. Mass ITN delivery integrated into a measles vaccination campaign with on-going ITN keep up through ANC resulted in 74% household ITN coverage two years after the campaign in the Lawra district of Ghana [8]. However, the most effective delivery strategy has yet to be determined and is likely to differ according to context. In particular, questions remain with regards to optimal delivery of ITNs through routine services.

For ITN delivery via routine services such as ANC, MCH and EPI to be successful in maintaining high and equitable coverage levels, it is necessary that a large proportion of the target population should be reached by the selected service(s), or should at least attend the delivery point. ANC and EPI coverage is variable in developing countries, especially in sub-Saharan Africa (SSA). The average coverage in SSA for at least one ANC visit is moderately high, 73%, but the range is very wide, from 28% to 98%. The coverage for DTP3 immunization among one year olds in the region ranges from 20% to 99%, with the average being 74% [12].

An association between attendance at ANC and at EPI would have implications for the development of effective and efficient delivery strategies. If women who attend ANC are the same women who take their child for EPI vaccinations, then such women are expected to receive an ITN through each system. Assuming that women sleep under the ITN during their pregnancy and the baby will share the bed once born [13], then only one ITN delivered through ANC may be required to protect both mother and child as long as they share a sleeping space. Conversely, if there are many women who attend one of these two services but not the other, then distribution through both ANC and EPI could make a valuable additional contribution to overall coverage, compared to the alternatives of distribution through just one channel (ANC but not EPI, or vice versa). Finally, there are women and children who would not gain protection through either of these channels because the mothers neither go to ANC nor take their children to EPI. Understanding the predictors of non-attendance will help programme managers target these particularly vulnerable women and their children for ITN delivery.

The current aim of ITN programmes is universal coverage and therefore the role of ANC and EPI delivery channels for providing sufficient ITNs to cover the whole household rather than just pregnant women and children may be considered. Only households that have a pregnant woman or a child under one would be reached through these routine systems.

Predictors and correlates of ANC or EPI attendance have been studied in many developing countries. For example, a recent systematic review to identify the factors of ANC utilization in developing countries included 28 studies set in various African, Asian, Middle Eastern, Central American and Caribbean countries [14]. Identified factors were placed in seven categories: (i) sociodemographic factors; (ii) availability; (iii) accessibility; (iv) affordability; (v) characteristics of health services; (vi) women's position in the household and society; and (vii) women's knowledge, attitudes, beliefs and culture. Numerous variables were identified, but maternal education, healthcare availability, residence and socioeconomic status were found to be strong predictors of ANC use. Other predictors found included: husband's education and occupation, parity, marital status, woman's age at pregnancy or marriage, ethnicity, religion, household size, travel required to health center, woman's occupation, exposure to mass media and woman's autonomy. The review also found that factors influencing ANC attendance are country specific [14]. Factors associated with initial, as well as complete, EPI attendance have also been studied in developing countries. For example, studies in Guinea and China found maternal education and cost to be strong predictors of initial EPI attendance, while age, household wealth and residence were less so [15,16]. Studies in Guinea, Ghana, and Nigeria found maternal education, employment, residence, mother's knowledge about vaccination and number of children to influence complete EPI attendance [15,17,18].

Although the characteristics of women who attend ANC and EPI have been investigated separately, the predictors of women attending both services, or attending neither have not yet been explored. National level data from three West African countries was used to explore the independence of ANC and EPI attendance amongst mothers of children under five years, and the predictors of those that do not attend either service.

## Methods

### Study populations

Chad, Mali and Niger were selected for the study because all three countries have ANC and EPI attendance below the regional averages of 73% (ANC) and 87% and 78% (DPT1 and DPT3), and also because malaria is a significant public health problem and recent relevant data is publicly available. ANC and EPI coverage in Chad and Niger are considerably lower than Mali, which has shown recent improvements in coverage [19]; in 2008 only 39% of women in Chad and 46% of women in Niger reported visiting ANC at least once, compared to 70% of women in Mali [12]. In 2008, Chad also had the lowest DPT1 and DTP3 coverage at 45% and 23%, respectively; Niger had the second lowest with 76% and 70%, while Mali had 85% and 74% DPT1 and DPT3 coverage [20]. Data from the 2004 Chad, 2006 Mali and 2006 Niger demographic and health surveys (DHS) were used for the analysis. The surveys were nationally representative and households were selected using a two-stage, stratified, clustered and not self-weighting sampling design. Few mothers shared a household, so clustering at the household level was not considered. Further details of the surveys can be found in the DHS final reports [19,21,22].

The analysis was restricted to the youngest living child for each woman at the time of the survey. ANC attendance was defined as at least one visit to a healthcare professional (doctor, nurse, or midwife) during their last pregnancy. EPI attendance was defined as receipt of first dose of the multivalent diphtheria, tetanus and pertussis vaccine (DTP1) amongst children 23 months of age, as confirmed by mother's report or a vaccination card. These definitions of attendance were used because only one visit to either service would be required for mothers to receive an ITN through routine distribution.

### Statistical analysis

Quantitative data was analyzed using STATA 11.0 (Stata Corporation, College Station, TX, USA) statistical package. Frequencies and proportions were used for the descriptive analysis. All analyses accounted for the design of the survey, adjusting for clustering and weighting.

The independence of attending both ANC and EPI was investigated using logistic regression (p ≤ 0.05). The number of women expected to attend both services, neither service or only one service if ANC and EPI attendance were independent was also calculated and compared to actual numbers observed using a two-sided z-test for difference between two proportions (without svy).

**Figure 1:**
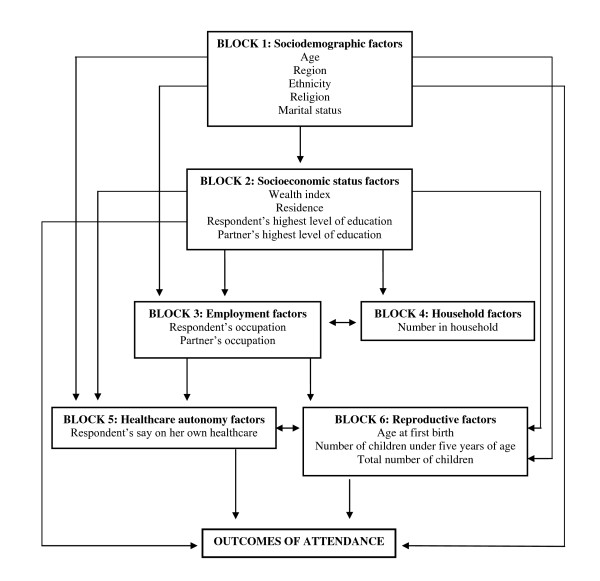
**Conceptual framework of potential predictors of ANC and EPI attendance**.

A range of sociodemographic and health related variables were selected to explore the potential predictors of women not attending either service. A basic conceptual framework was developed before conducting the univariate analysis, to explore how hierarchical relationships between these variables may influence negative attendance. Although frameworks exist for treatment seeking for infectious diseases [23-25], no such frameworks relating specifically to preventative health care-seeking behavior were identified. Therefore, the present framework was created as an expansion of these existing frameworks, based around six individual blocks (Figure 1): (i) Sociodemographic factors (age, region, religion, ethnicity, marital status); (ii) Socioeconomic status factors (wealth index, residence, mother's highest education level, partner's highest education level); (iii) Employment factors (mother's occupation, partner's occupation); (iv) Household factors (household size); (v) Healthcare autonomy (respondent's final say on her own healthcare); and (vi) Reproductive factors (respondent's age at birth of first child, total number of children born, number of children under five years of age).

Preliminary univariate predictors of attendance were determined by a Wald test p-value of < 0.10 using logistic regression. Multivariate adjusted odds ratios (OR) and 95% confidence intervals (95% CI) were calculated to determine the final predictors of non-attendance at both ANC and EPI (p ≤ 0.05).

### Venn diagrams

Venn diagrams were created using BioInfoRx area-proportional Venn diagram plotter and editor http://bioinforx.com/free/bxarrays/venndiagram.php.

### Ethical approval

Ethical approval for the analysis was obtained from the Ethics Committee of the London School of Hygiene and Tropical Medicine. Permission to use the datasets for this analysis was granted from Measure DHS.

## Results

### Chad

**Figure 2:**
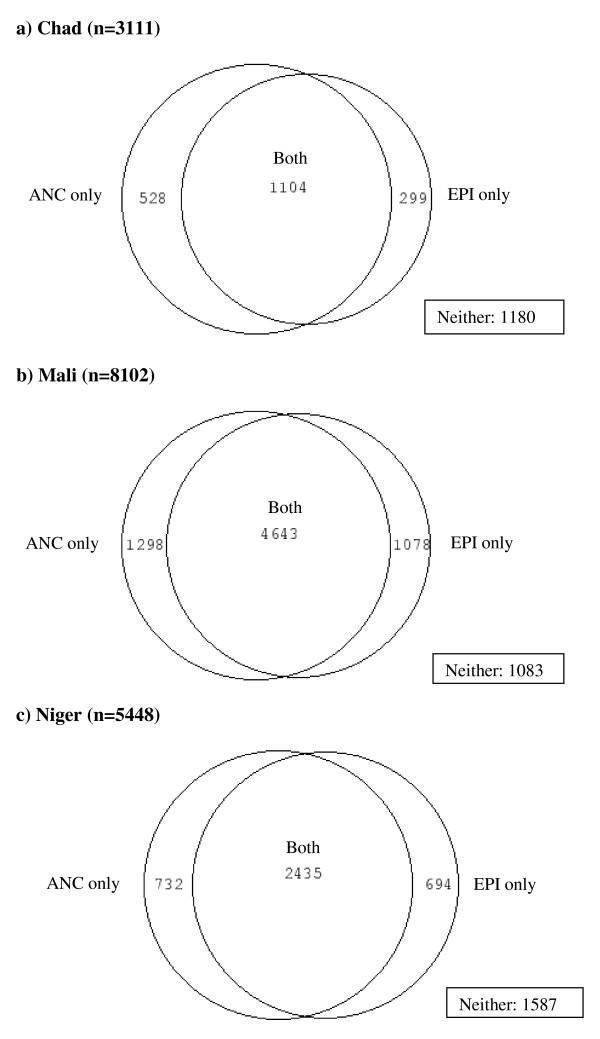
**Venn diagrams of ANC and EPI attendance in a) Chad; b) Mali; and c) Niger**.

In Chad, 3, 507 mothers between 15 and 49 years of age were sampled. Half of the mothers were between 20 and 29 years old and 81% lived in rural areas. Approximately 46% were from southern regions, 77% worked in agriculture and 77% had no education. Only 43% of women interviewed attended ANC at least once during their most recent pregnancy; 36% of women had their youngest child vaccinated against DTP1. Forty-seven percent of women did not attend either service with their youngest child (Figure 2a). Of the women who attended neither ANC nor EPI, 32% of women were in the poorest wealth quintile, 82% worked in agriculture, 93% had no education, 50% were between 20 and 29 years old and 30% were from southern regions.

ANC and EPI attendance were strongly associated in Chad. Mothers who attended ANC were over six times more likely to attend EPI with their child than those who did not attend ANC (OR 6.3, 95% CI 4.9-8.1). The observed number who did not attend ANC or EPI (1180) varied from the number expected (812) to be non-attendees if attendance was independent (p < 0.0001). Similarly, the observed number of mothers who attended both services (1104) differed from the expected number (736) if attendance was independent (p < 0.0001) (Table 1; Figure 2a).

**Table 1:** Actual and expected numbers of attendees if attendance was independent in Chad.

	Attended EPI ***(expected)***	P-value	Did not attend EPI ***(expected)***	P-value	Total
**Attended ANC** ***(expected)***	1104 *(736)*	< 0.0001	528 *(896)*	< 0.0001	1632

**Did not attend ANC** ***(expected)***	299 *(667)*	< 0.0001	1180 *(812)*	< 0.0001	1479

**Total**	1403		1708		3111

Of the initial 15 variables chosen for the exploratory analysis (there was no variable available for healthcare autonomy), age, marital status, household size and age at the first birth were not significantly associated with attending neither service (p ≥ 0.1). Region, residence, education, occupation, religion, ethnicity and wealth index were found to be strong predictors (p < 0.0001) in the univariate analysis (Table 2).

**Table 2:** Predictors of women not attending ANC or EPI in Chad (n = 1180), adjusted for clustering.

Variable	Attended neither % (n)	Unadjusted OR (95% CI)	Wald	Adjusted OR (95% CI)	Wald
			p-value		p-value
**Region**			< 0.0001		0.001
South	31.3 (263)	1.0		1.0	
East	79.6 (341)	8.5 (4.9-15.0)		2.6 (1.3-5.0)	
North	51.4 (167)	2.3 (1.2-4.5)		1.1 (0.5-2.3)	
Central	59.8 (142)	3.3 (1.5-7.0)		1.0 (0.4-2.6)	
West	50.3 (267)	2.2 (1.4-3.6)		2.4 (0.3-4.4)	

**Residence**			< 0.0001		< 0.0001
Rural	54.5 (991)	1.0		1.0	
Urban	12.6 (189)	0.1 (0.08-0.2)		0.2 (0.1-0.3)	

**Mother's education**			< 0.0001		< 0.0001
None	55.9 (1110)	1.0		1.0	
Primary	16.8 (62)	0.2 (0.1-0.2)		0.4 (0.3-0.6)	
Secondary	6.3 (8)	0.1 (0.02-0.1)		0.4 (0.2-1.0)	
Higher	0 (0)	---		---	

**Partner's education**			< 0.0001		< 0.0001
None	60.0 (998)	1.0		1.0	
Primary	31.2 (128)	0.3 (0.2-0.4)		0.7 (0.5-1.0)	
Secondary	11.7 (48)	0.1 (0.05-0.1)		0.3 (0.2-0.4)	
Higher	0.9 (1)	0.01 (0.001-0.05)		---	

**Partner's occupation**			< 0.0001		0.004
Professional	50.3 (1041)	1.0		1.0	
Unemployed	17.3 (11)	0.2 (0.1-0.5)		1.5 (0.5-4.5)	
Agriculture/manual labour	28.4 (117)	0.4 (0.3-0.5)		0.6 (0.4-0.8)	
Clerical, sales, services	16.6 (3)	0.2 (0.1-0.6)		1.0 (0.4-2.2)	

**Number of children under 5**			0.08		0.001
1	47.1 (1126)	1.0		1.0	
2	37.2 (33)	0.7 (0.4-1.2)		1.2 (0.6-2.5)	
3	14.5 (1)	0.2 (0.05-0.8)		0.2 (0.1-0.4)	

**Wealth index**			< 0.0001		0.1
Poorest	82.2 (432)	1.0		1.0	
Second	50.8 (242)	0.2 (0.1-0.3)		0.7 (0.4-1.0)	
Middle	45.3 (179)	0.2 (0.1-0.3)		0.6 (0.4-1.0)	
Fourth	38.0 (178)	0.1 (0.1-0.2)		0.5 (0.3-0.9)	
Least poor	16.8 (149)	0.04 (0.02-0.1)		0.4 (0.2-0.8)	

**Ethnicity**			< 0.0001		0.2
Others	50.9 (694)	1.0		1.0	
Arabic	66.4 (253)	1.9 (1.3-2.9)		1.0 (0.6-1.6)	
Kanem-bornou	47.8 (136)	0.9 (0.5-1.5)		0.6 (0.3-0.9)	
Sara	26.1 (97)	0.3 (0.2-0.6)		0.9 (0.5-1.7)	

**Religion**			< 0.0001		0.2
Muslim	59.2 (954)	1.0		1.0	
Catholic	30.9 (118)	0.3 (0.2-0.5)		0.5 (0.3-1.0)	
Protestant	25.8 (69)	0.2 (0.1-0.4)		0.5 (0.3-1.0)	
Other	42.7 (38)	0.5 (0.3-1.0)		0.7 (0.3-1.4)	

**Mother's occupation**			< 0.0001		0.2
Agriculture/manual labour	25.7 (109)	1.0		1.0	
Unemployed	40.1 (264)	1.9 (1.2-3.0)		1.3 (0.9-2.0)	
Clerical, sales, services	0 (0)	---		---	
Professional	53.3 (803)	3.3 (2.1-5.3)		1.4 (0.9-2.1)	

Residence and the education level of mothers and partners remained very strong predictors of non-attendance at both ANC and EPI after controlling for the other variables in the multivariate model (p < 0.0001). Region (p = 0.001), partner's occupation (p = 0.002) and number of children under five (p = 0.001) were also found to be strong predictors. Wealth index, ethnicity, religion and mother's occupation were not predictors (p > 0.05) (Table 2).

Women living in eastern and western regions of Chad were almost three times as likely to not attend either service compared to women in southern regions (OR 2.7, 95% CI 1.3-5.0; OR 2.5, 95% CI 1.4-4.5). Mothers who had three children under five years of age were 80% less likely to be non-attendees than women who had only one child younger than five (OR 0.2, 95% CI 0.1-0.4). Mothers educated to a primary school level had reduced odds of non-attendance (OR 0.4, 95% CI 0.3-0.6), however, those with a secondary school education may not have lower odds of non-attendance compared to women with no education at all (OR 0.4, 95% CI 0.2-1.0). Odds of non-attendance were lower in urban areas (OR 0.2, 95% CI 0.1-0.3). Women had lower odds of non-attendance if their partner worked in agriculture compared to those who were professionals (OR 0.5, 95% CI 0.4-0.7). However, the odds of non-attendance decreased as their partner's education level increased (Table 2).

### Mali

The Mali study population consisted of 9, 036 mothers aged between 15 and 49 years. Almost half of the mothers were between 20 and 29 years and 71% lived in rural areas. Only 9% of women were from northern and eastern regions, while the rest of the study population was split evenly between western, central and southern regions. Only 0.5% of women had professional, technical or managerial jobs, while the rest were either unemployed, worked in agriculture or manual labor or clerical, sales or services and 84% of women had no education. Sixty-nine percent of mothers attended ANC at least once during their last pregnancy and 70% had their youngest child vaccinated against DTP1. Only 12% of mothers did not attend ANC or EPI with their youngest child (Figure 2b). Of these women, nearly 95% had no education, roughly 50% were in the bottom two wealth quintiles, 48% were unemployed and 38% lived in western regions.

ANC and EPI attendance were strongly associated in Mali. Mothers who attended ANC were over three times more likely to attend EPI with their child than those who did not attend ANC (OR 3.2, 95% CI 2.7-3.9). The observed number who did not attend ANC or EPI (1083) was higher than the number expected (635) to be non-attendees if attendance was independent (p < 0.0001). Similarly, the observed number of mothers who attended both services (4643) was higher than the expected number (4195) if attendance was independent (p < 0.0001) (Table 3; Figure 2b).

**Table 3:** Actual and expected numbers of attendees if attendance was independent in Mali.

	Attended EPI ***(expected)***	P-value	Did not attend EPI ***(expected)***	P-value	Total
**Attended ANC** ***(expected)***	4643 *(4195)*	< 0.0001	1078 *(1526)*	< 0.0001	5721

**Did not attend ANC** ***(expected)***	1298 *(1746)*	< 0.0001	1083 *(635)*	< 0.0001	2381

**Total**	5941		2161		8102

Marital status, total number of children and number of children under five were eliminated as potential predictors at the univariate stage (p ≥ 0.1). Region, residence, wealth index, ethnicity, education and mother's occupation were very strong predictors of attending neither ANC nor EPI in the univariate analysis (p < 0.0001). Partner's occupation (p = 0.0004) was also found to be a strong predictor, while healthcare autonomy (p = 0.07), household size (p = 0.04), and age at first birth (p = 0.05), religion (p = 0.06) and age (p = 0.07) were less so (Table 4).

**Table 4:** Predictors of women not attending ANC or EPI in Mali (n = 1083), adjusted for clustering.

Variable	Attended neither % (n)	Unadjusted OR (95% CI)	Wald	Adjusted OR (95% CI)	Wald
			p-value		p-value
**Region**			< 0.0001		0.0002
Western	14.6 (298)	1.0		1.0	
Central	11.5 (265)	0.8 (0.5-1.1)		0.7 (0.5-1.2)	
Eastern	20.6 (184)	1.5 (0.8-2.7)		1.4 (0.7-2.8)	
Northern	30.4 (174)	2.5 (1.5-4.4)		2.0 (1.1-3.7)	
Southern	6.5 (162)	0.4 (0.2-0.7)		0.6 (0.3-1.0)	

**Wealth index**			< 0.0001		0.03
Poorest	14.5 (221)	1.0		1.0	
Second	16.3 (301)	1.3 (1.1-1.5)		1.0 (0.7-2.8)	
Middle	17.0 (344)	1.4 (1.1-1.7)		1.0 (0.7-1.3)	
Fourth	10.2 (184)	0.7 (0.5-0.8)		0.7 (0.5-1.0)	
Least poor	2.7 (33)	0.1 (0.09-0.2)		0.4 (0.2-0.8)	

**Ethnicity**			< 0.0001		0.001
Other	14.2 (658)	1.0		1.0	
Bambara	8.3 (152)	0.6 (0.4-0.8)		0.6 (0.4-0.9)	
Peulh	16.4 (180)	1.2 (0.9-0.6)		1.1 (0.8-1.6)	
Sarkole	10.2 (92)	0.7 (0.4-1.1)		0.7 (0.4-1.1)	

**Mother's education**			< 0.0001		0.04
None	13.8 (1020)	1.0		1.0	
Primary	5.2 (52)	0.4 (0.3-0.5)		0.5 (0.3-0.9)	
Secondary	1.8 (11)	0.1 (0.07-0.2)		0.3 (0.1-1.0)	
Higher	0 (0)	---		---	

**Partner's education**			< 0.0001		0.05
None	14.0 (979)	1.0		1.0	
Primary	7.7 (59)	0.5 (0.4-0.7)		0.7 (0.5-1.0)	
Secondary	5.1 (26)	0.3 (0.1-1.1)		0.8 (0.4-1.6)	
Higher	0.7 (2)	0.2 (0.1-0.6)		0.2 (0.02-2.4)	
Don't know	3.5 (5)	0.8 (0.3-2.6)		0.3 (0.1-0.9)	

**Mother's occupation**			< 0.0001		0.001
Agriculture/manual labour	14.4 (461)	1.0		1.0	
Unemployed	13.4 (520)	1.0 (0.7-1.3)		1.0 (0.8-1.4)	
Clerical, sales, services	5.6 (93)	0.4 (0.2-0.5)		0.6 (0.4-0.9)	
Professional	0 (0)	---		---	
Other	52.5 (1)	6.6 (0.6-78.1)		23.0 (2.5-209.4)	
Don't know	3.5 (6)	0.2 (0.1-0.9)		0.3 (0.1-1.5)	

**Age**			0.07		0.06
15-19	14.9 (123)	1.0		1.0	
20-29	11.7 (514)	0.8 (0.6-1.0)		0.8 (0.6-1.0)	
30-39	12.7 (351)	0.8 (0.6-1.1)		0.8 (0.6-1.1)	
40-49	10.3 (95)	0.7 (0.5-0.9)		0.6 (0.4-0.9)	

**Residence**			< 0.0001		0.1
Rural	15.2 (970)	1.0		1.0	
Urban	5.0 (113)	0.3 (0.2-0.5)		0.7 (0.5-1.1)	

**Religion**			0.06		0.3
Muslim	12.4 (1007)	1.0		1.0	
Christian	6.4 (21)	0.5 (0.3-0.9)		0.6 (0.3-1.2)	
None	11.2 (28)	0.9 (0.5-1.7)		0.9 (0.5-1.6)	
Other	15.2 (21)	1.3 (0.7-2.1)		1.3 (0.8-2.3)	

**Partner's occupation**			0.0004		0.7
Agriculture/manual labour	13.6 (884)	1.0		1.0	
Clerical, sales, services	8.4 (114)	0.6 (0.4-0.9)		1.0 (0.7-1.5)	
Professional	7.8 (13)	0.5 (0.1-2.8)		1.8 (0.3-9.1)	
Other	12.2 (25)	0.9 (0.5-1.5)		1.0 (0.6-1.8)	
Don't know	3.4 (24)	0.4 (0.2-0.8)		0.7 (0.4-1.2)	

**Healthcare autonomy**			0.02		0.3
Husband/partner only	12.7 (850)	1.0		1.0	
Respondent alone	10.8 (100)	0.8 (0.6-1.2)		1.0 (0.7-1.4)	
Respondent & husband	8.8 (46)	0.7 (0.4-1.1)		0.8 (0.5-1.2)	
Respondent & other	5.0 (1)	0.4 (0.05-2.6)		0.8 (0.1-6.5)	
Someone else	11.7 (80)	0.9 (0.6-1.3)		1.1 (0.8-1.6)	
Other	41.9 (5)	5.0 (1.4-17.4)		4.7 (1.1-20.2)	

**Age at first birth**			0.05		0.09
11-12	12.9 (17)	1.0		1.0	
13-19	12.7 (768)	1.0 (0.5-1.8)		1.2 (0.6-2.7)	
20-29	10.5 (280)	0.8 (0.4-1.5)		1.1 (0.5-2.3)	
30-39	20.3 (18)	1.7 (0.7-4.0)		2.3 (0.9-6.1)	
40-49	0 (0)	---		---	

**Household size**			0.04		0.1
1-5	13.1 (469)	1.0		1.0	
6-10	12.7 (496)	1.0 (0.8-1.2)		1.1 (0.8-1.4)	
11+	9.2 (118)	0.7 (0.5-1.0)		0.8 (0.5-1.3)	

Region (p = 0.0002), ethnicity (p = 0.001) and mother's occupation (p = 0.001) remained strong predictors of non-attendance in the multivariate model, while wealth index (p = 0.03), mother's education (p = 0.04) and partner's education (0.05) were less so (Table 4).

Malian mothers who lived in northern regions had twice the odds of non-attendance than those who lived in western regions after adjusting for the other possible predictors (OR 2.0, 95% CI 1.1-3.7). The Bambara ethnic group had reduced odds of non-attendance compared to ethnic minorities grouped together (OR 0.6, 95% CI 0.4-0.9). Women in the highest wealth quintile were 60% less likely to be non-attenders compared to women in the lowest quintile (OR 0.4, 95% CI 0.2-0.8). Mothers with a primary level of education had half the odds of non-attendance when compared to women with no education at all (OR 0.5, 95% CI 0.3-0.9). Furthermore, women working in clerical, sales or services had reduced odds of non-attendance compared to unemployed mothers (OR 0.6, 95% CI 0.4-0.9) (Table 4).

### Niger

In Niger, 5, 884 mothers between 15 and 49 years of age were sampled. Forty-five percent of women were between 20 and 29 years and 85% lived in rural areas. Forty percent were from southern regions of Niger, while only 5% were from northern or eastern regions. Over half of the mothers were unemployed and 87% had no education. Forty-seven percent of mothers attended ANC at least once with their youngest child. Fifty-one percent of the mothers had their youngest child vaccinated against DTP1; 36% of mothers did not attend either service with her youngest child (Figure 2c). Of these women, 55% were from southern regions of Niger, 56% were unemployed and 28% belonged to the lowest wealth quintile. Nearly 95% of women who did not attend either service had no education.

ANC and EPI attendance were strongly associated in Niger. Mothers who attended ANC were nearly seven times more likely to attend EPI with their child than those who did not attend ANC (OR 6.7, 95% CI 5.5-8.1). The observed number who did not attend ANC or EPI (1587) was higher than the number expected (971) to be non-attendees if attendance was independent (p < 0.0001). Similarly, the observed number of mothers who attended both services (2435) was higher than the expected number (1819) if attendance was independent (p < 0.0001) (Table 5; Figure 2c).

**Table 5:** Actual and expected numbers of attendees if attendance was independent in Niger.

	Attended EPI ***(expected)***	P-value	Did not attend EPI ***(expected)***	P-value	Total
**Attended ANC** ***(expected)***	2435 *(1819)*	< 0.0001	694 *(1310)*	< 0.0001	3129

**Did not attend ANC** ***(expected)***	732 *(1348)*	< 0.0001	1587 *(971)*	< 0.0001	2319

**Total**	3167		2281		5448

All variables included in the univariate analysis were associated with non-attendance except mother's current age (p = 0.52) and number of children under five (p = 0.83). Region, partner's occupation, education, wealth index, ethnicity and residence were found to be very strong predictors at this stage (p < 0.0001). Healthcare autonomy (p = 0.0003) and religion (p = 0.01) were also found to be strong univariate predictors (Table 6).

**Table 6:** Predictors of women not attending ANC or EPI in Niger (n = 1587), adjusted for clustering.

Variable	Attended neither % (n)	Unadjusted OR (95% CI)	Wald	Adjusted OR (95% CI)	Wald
			p-value		p-value
**Region**			< 0.0001		< 0.0001
Southern	49.5 (640)	1.0		1.0	
Northern	18.4 (71)	0.2 (0.1-0.4)		0.3 (0.2-0.6)	
Eastern	35.3 (144)	0.6 (0.3-0.9)		0.3 (0.2-0.6)	
Western	23.3 (448)	0.3 (0.2-0.4)		0.4 (0.3-0.7)	
Central	32.5 (284)	0.5 (0.3-0.8)		0.4 (0.3-0.7)	

**Wealth index**			< 0.0001		< 0.0001
Poorest	47.3 (465)	1.0		1.0	
Second	43.2 (347)	0.9 (0.7-1.1)		0.9 (0.7-1.1)	
Middle	40.6 (322)	0.8 (0.6-1.0)		0.8 (0.6-1.0)	
Fourth	38.2 (348)	0.7 (0.5-0.9)		0.8 (0.5-1.1)	
Least poor	9.0 (105)	0.1 (0.08-0.02)		0.2 (0.2-0.4)	

**Residence**			< 0.0001		0.004
Rural	41.3 (1464)	1.0		1.0	
Urban	8.2 (123)	0.1 (0.08-0.2)		0.5 (0.3-0.8)	

**Ethnicity**			< 0.0001		< 0.0001
Hausa	35.9 (712)	1.0		1.0	
Djerma	16.9 (183)	0.4 (0.2-0.6)		0.6 (0.4-0.9)	
Tuareg	48.7 (295)	1.7 (1.2-2.3)		1.7 (1.1-1.6)	
Other	52.2 (396)	1.9 (1.3-2.8)		2.1 (1.4-3.3)	

**Religion**			0.01		0.04
Muslim	35.7 (1548)	1.0		1.0	
Christian	48.4 (13)	1.7 (0.8-3.4)		1.7 (0.9-3.2)	
None	69.4 (35)	4.1 (1.7-10.0)		3.0 (1.1-8.11)	
Other	0 (0)	---		---	

**Mother's education**			< 0.0001		0.001
None	39.3 (1508)	1.0		1.0	
Primary	19.1 (77)	0.4 (0.3-0.5)		0.6 (0.4-0.9)	
Secondary	0.1 (2)	0.02 (0.003-0.1)		0.1 (0.01-0.6)	
Higher	0 (0)	---		---	

**Partner's education**			< 0.0001		< 0.0001
None	40.3 (1464)	1.0		1.0	
Primary	19.5 (80)	0.4 (0.3-0.5)		0.5 (0.3-0.7)	
Secondary	7.3 (19)	0.1 (0.06-0.2)		0.3 (0.1-0.5)	
Higher	0 (0)	---		---	
Don't know	15.9 (16)	0.3 (0.1-0.6)		0.6 (0.2-1.6)	

**Household size**			0.03		0.03
1-5	38.4 (517)	1.0		1.0	
6-10	37.2 (735)	1.0 (0.8-1.1)		0.9 (0.7-1.1)	
11-15	41.7 (230)	0.7 (0.5-0.9)		0.8 (0.6-1.0)	
16-20	48.6 (60)	0.7 (0.5-1.2)		1.4 (0.9-2.2)	
21+	40.8 (45)	0.7 (0.4-1.3)		1.6 (0.9-2.9)	

**Healthcare autonomy**			0.0003		0.04
Husband/partner only	38.9 (1186)	1.0		1.0	
Respondent alone	28.9 (237)	0.6 (0.4-0.8)		0.8 (0.6-1.1)	
Respondent & husband	26.9 (84)	0.6 (0.4-0.8)		0.7 (0.5-1.0)	
Respondent & other	19.9 (8)	0.4 (0.2-0.9)		0.6 (0.2-1.4)	
Someone else	33.7 (65)	0.8 (0.5-1.2)		1.1 (0.7-1.7)	
Other	12.5 (5)	0.2 (0.07-0.7)		0.3 (0.1-0.9)	

**Marital status**			0.05		0.3
Currently married	36.3 (1542)	1.0		1.0	
Never married	21.0 (5)	0.5 (0.2-1.3)		---	
Formerly married	26.0 (40)	0.6 (0.4-1.0)		0.8 (0.4-1.3)	

**Mother's occupation**			0.03		0.06
Agriculture/manual labour	37.5 (426)	1.0		1.0	
Unemployed	37.8 (942)	1.0 (0.8-1.3)		1.3 (1.0-1.7)	
Clerical, sales, services	30.0 (213)	0.7 (0.5-1.0)		0.9 (0.7-1.3)	
Professional, army	0 (0)	---		---	
Other	51.6 (3)	1.8 (0.3-12.3)		3.0 (0.3-27.4)	

**Partner's occupation**			< 0.0001		0.7
Agriculture/manual labour	41.3 (1117)	1.0		1.0	
Unemployed	8.0 (2)	0.1 (0.03-0.6)		1.0 (0.1-8.9)	
Clerical, sales, services	27.7 (388)	0.5 (0.4-0.7)		0.9 (0.7-1.1)	
Professional, army	5.4 (11)	0.1 (0.02-0.3)		1.5 (0.5-4.6)	
Other	39.0 (56)	0.9 (0.5-1.6)		0.9 (0.5-1.7)	

**Age at first birth**			0.02		0.9
12	34.5 (12)	1.0		1.0	
13-19	37.4 (1224)	1.1 (0.5-2.5)		1.0 (0.4-2.6)	
20-29	31. (342)	0.9 (0.4-1.9)		1.0 (0.4-2.8)	
30-39	39.3 (9)	1.2 (0.4-3.9)		1.7 (0.4-7.7)	
40-49	0 (0)	---		---	

**Total number of children**			0.03		0.3
1-3	32.9 (613)	1.0		1.0	
4-6	38.5 (564)	1.3 (1.1-1.5)		1.2 (1.0-1.4)	
7-9	38.3 (306)	1.3 (1.0-1.6)		1.1 (0.9-1.4)	
10+	35.7 (104)	1.1 (0.8-1.5)		0.9 (0.7-1.3)	

Wealth index, region, ethnicity, residence and both mother's and partner's highest education level remained very strong predictors of not attending either service, after adjusting for the other possible predictors in the multivariate model (p < 0.0001). Healthcare autonomy (p = 0.0003), religion (p = 0.01) and household size (p = 0.04) were also found to be predictors in the multivariate model (Table 6).

Nigerien mothers living in northern, eastern, western and central regions were less likely to be non-attenders than mothers in southern regions. Those in the north and east were 70% less likely (OR 0.3, 95% CI 0.2-0.6), while those in western and central regions were 60% less likely (OR 0.4, 95% CI 0.3-0.7) to not attend. Women in urban areas were half as likely to not attend ANC or EPI compared to those living in rural areas (OR 0.5, 95% CI 0.3-0.8). The odds of not attending ANC or EPI increased as household size increased. The odds of attending neither service were three times higher in women who did not practice a religion compared to Muslim women (OR 3.0, 95% CI 1.1-8.11). Mothers of Djerma ethnicity were 40% less likely to be non-attenders than Hausan (OR 0.6, 95% CI 0.4-0.9). Tuareg mothers were almost twice as likely to not attend either service (OR 1.8, 95% CI 1.1-1.6), while other ethnic minorities combined had over twice the odds of non-attendance (OR 2.1, 95% CI 1.4-3.3) (Table 6).

Interaction between wealth index and region was explored for all three countries, however, it was not found to be significant (p > 0.05) and was not included in the final multivariate model.

## Discussion

Routine services, such as ANC and EPI, have been shown to be successful points of ITN access for those population groups most biologically vulnerable to malaria [8,10]. However, questions remain on whether delivery should be through either EPI or ANC or both. This is likely to involve a balance between effectiveness and equity (i.e. ensuring the most vulnerable populations are reached by the intervention) and efficiency (i.e. avoiding excessive duplication of resources). Key factors that will influence such decisions on strategy for routine delivery of ITNs in any particular country are: (i) the proportion of pregnant women attending ANC; (ii) the proportion of children attending at least one EPI session; (iii) whether attendance of these two services is independent of each other; (iv) the effectiveness of the delivery processes via each of these services; and (v) the cost effectiveness of delivery through each of these services independently and in combination.

In this paper, the independence of ANC attendance and EPI attendance was investigated, and the predictors of mothers not attending either ANC or EPI for Chad, Mali and Niger, three West African countries with lower than average ANC and EPI coverage were identified. This is the first analysis examining the predictors of non-attendance for routine ITN delivery purposes. Alternative routine delivery systems need to be considered for women who do not attend ANC or EPI.

Forty-seven percent of women in Chad, 12% in Mali and 36% in Niger did not attend ANC or EPI with their youngest child. The findings show that attendance at ANC and attendance at EPI are not independent, that is women that attend ANC are more likely to take their child to EPI than women that do not attend ANC, or conversely children taken to EPI are more likely to have mothers that attended ANC. This means that there is a greater degree of overlap of women and children attending both services than would be predicted if attendance was independent. In terms of ITN delivery, this indicates there would be a considerable proportion of women who would receive two ITNs if delivery was through both ANC and EPI. Although attendance at ANC and EPI are not independent, the attendance figures from Chad, Mali and Niger seems to suggest that delivery through both systems may still add incrementally to delivery through one alone. Therefore, there is still potential to increase the proportion of women and children receiving ITNs by delivering through both of these channels. For example, if ITNs were distributed through EPI as well as ANC in Chad, Mali or Niger, an additional 10.6%, 16.1% or 16.0% of children would receive an ITN, respectively (Tables 1, 3, 5; Figure 2). Balancing the additional resources required to deliver ITNs through both EPI and ANC with the potential gain achievable in terms of additional children covered is a dilemma for programme managers. Cost effectiveness modelling and analysis of the determinants of household level ITN allocation are required to aid in this decision making in order to cover as many households as possible. However, the level of overlap in a country with high ANC and EPI coverage may be an efficiency issue and the relative additional proportion of households covered by ITNs delivered through ANC and EPI may not be as beneficial as designing an alternative strategy to reach the non-attenders.

The predictors of attending neither ANC nor EPI were similar for Chad, Mali and Niger. Region and mother's and partner's highest level of education attained were predictors in all three countries. The odds of non-attendance decreased as the level of education for mothers and their partners increased. Women in northern regions of Mali were twice as likely not to attend either ANC or EPI compared to women in western regions. Some ethnic groups in these regions are isolated due to geography and nomadism, so it may be difficult for them to access ANC and EPI services [26]. In Niger, political and ethnic unrest has been common in northern and eastern regions, while a large portion of the country was plagued with drought and famine in 2005 and 2006, around the time of the survey. Despite this, mothers in northern, eastern, western and central regions had much lower odds of non-attendance than mothers in southern regions, which may be explained by a sudden influx of medical aid due to these conditions. Mothers in eastern and western regions of Chad were over twice as likely to be non-attendees compared to those in southern regions. Poverty, conflict and refugee camps are common in these regions as Chad borders Sudan and the Central African Republic [27].

Wealth index and ethnicity were predictors in Mali and Niger. The odds of non-attendance decreased as wealth index increased, although in Mali this effect of wealth index was seen only in the richest two quintiles. Women in the higher socioeconomic groups are most likely able to afford the services, and are likely to also be more educated and understand the importance of preventive health interventions such as antenatal care and vaccination. Mothers in Niger of Djerma ethnicity were less likely to be non-attenders, while Tuareg and other ethnic minorities had increased odds of non-attendance. Increased child mortality relating to ethnic inequalities has been observed in SSA, where reduced odds of child mortality in the Djerma have been reported [28]. Occupation was also a predictor in Mali and Niger. In Mali, mothers who held clerical, sales or service positions had lower odds of non-attendance compared to women working in agriculture or manual labour. This may be because of improved financial capacity or perhaps due to higher education levels necessary to hold such an occupation, increasing awareness that these services are crucial to their child's health. Interestingly, mothers in Chad whose partners worked in agriculture had lower odds of non-attendance than if their partner was a professional.

Some factors were predictors in one or two, but not all three countries. These included number of children under five years old in Chad, and household size, healthcare autonomy and religion in Niger. Chadian mothers with three children under five were 80% less likely to not attend either service; this may be because they had experienced benefits from previously attending ANC or EPI with their older children. Decreased healthcare autonomy was a predictor for women in Niger that did not attend ANC or EPI. Women who made their own decisions, or were involved in the process were less likely to be non-attendees compared to women whose partners made these decisions without them. Similarly, lack of autonomy has been found to be an important barrier to attending ANC in Asia [14]. Mothers with no religious affiliation were three times more likely than Muslim women to not attend either service in Niger. Muslim women have been reported to seek maternal health services more often than other religions in India and Ethiopia [29,30]. It is not clear if traditional animist beliefs were defined as no religion in the survey, in which case there have been similar reports of low ANC attendance among animist women in Ethiopia [30].

Although the WHO recommends universal coverage of 80% of all populations at risk with an insecticide-treated net, special attention must still be given to biologically vulnerable populations [31]. Chad, Mali and Niger have all adopted an ITN policy that includes continuous, free distribution to pregnant women and under-fives through ANC and EPI. In addition to their policies of routine ITN delivery, there are plans to deliver at least 2.9 million ITNs in Chad by the end of October 2011 and at least 6.5 million ITNs by early 2012 in Mali [32]. In 2007, an integrated child health campaign in Mali delivered 2.4 million ITNs and brought household ownership to 88% [33]. In more recent years, a total of 1.3 million ITNs in Chad and seven million ITNs in Mali were delivered through targeted campaigns by the end of 2010 [34,35]. A national integrated campaign was conducted in Niger in 2006 [7] and approximately 1.7 million ITNs were delivered in 2010 [36].

The latest national level survey data shows 27% of under-fives in Chad slept under any net the night before the survey and ITN use was only 1% (no data available for pregnant women) [37]. Forty-one percent of Malian under-fives slept under any net and 27% slept under an ITN, while 29% of pregnant women used an ITN [19]. In Niger, any net use was 15% among under-fives with 7% using an ITN, and 7% of pregnant women slept under an ITN [22]. The planned campaigns will go some way to improving the currently low ITN ownership levels in these countries via "catch up".

Forty-seven percent of women in Chad, 12% in Mali and 36% in Niger did not attend ANC or EPI with their youngest child. It is clear that ANC and EPI attendance in these countries must increase to ensure that pregnant women and their children receive proven preventive interventions provided at these delivery points, including "keep up" with ITNs. This analysis highlights the characteristics of women who are missed by routine services; these are the women who it is important to reach with mass ITN campaigns in the short term, and also interventions to improve ANC and EPI attendance in the longer term. However, increasing routine service coverage isn't a quick fix as it involves strengthening the health system in addition to improving demand, a well-known challenge in most SSA countries due to geographic, financial and political reasons [38].

Interestingly, there is evidence from other countries that integration of ITN delivery into routine services increases attendance. For example, an ITN voucher delivery system, including community mobilization activities, in Tanzania resulted in 97% of women attending a MCH clinic [39]. Furthermore, integration into EPI resulted in full vaccine schedule attendance increasing by 29% and 54% in two regions of Malawi [40]. This may provide further support for the distribution of ITNs through routine services in countries where current attendance is low, although is unlikely to solve all of the issues surrounding low attendance.

However, steps must be taken to increase ITN use not just ownership [41]. The factors contributing to the gap between ownership and use are not always clear. At a minimum, behaviour change communication must be provided to inform women about the importance of ITNs, proper use and maintenance. For example, one study found that mothers in Niger knew that under-fives were at risk of malaria, but were not aware of the protection offered by an ITN or that pregnant women were also at high risk [7].

It is important to note that the most recent available national level data was used for this analysis, although in many cases it was limited and several years old; in particular updated ITN ownership and use figures after the planned campaigns in Chad and Mali may show more encouraging results. Recall bias may have been a problem within the surveys as EPI attendance was defined by a vaccination card or mother's report and information on ANC attendance relates to the woman's most recent pregnancy which could be five to eight years prior to the survey. However, these are standard indicators used and still present the best objective estimates of ANC and EPI coverage. Statistically, some calculations were based on few observations although variables (such as ethnicity and region) were regrouped as finely as possible to limit this.

Region and education were identified as predictors of not attending ANC or EPI in Chad, Mali and Niger; additional country specific predictors included urban/rural residence, wealth index, ethnicity, religion, healthcare autonomy, household size, number of children under five and occupation. These are the groups of women that must be reached during ITN campaigns in order to achieve current coverage targets and cover pregnant women and under-fives in an equitable manner. In addition, interventions to increase attendance of ANC and EPI by these groups (and overall), and the effective delivery of ITNs through these channels have multiple potential benefits in terms of sustaining ITN coverage levels and maternal and child health.

This analysis focused on three West African countries; however, further studies are needed to establish predictors of non-attendance in different countries and regions to explore where differences in culture, views on healthcare and health system structure may have implications for ANC and EPI attendance and therefore on the best ITN delivery strategy to reach universal coverage of vulnerable groups. This analysis will be most important where attendance of both ANC and EPI is low. If attendance at either is approaching 100% then that would be the system of choice and the other would not be needed. There is scope for modelling of an attendance cut-off point where both are needed or just one is needed. Additionally, further research is needed to identify the age at which children no longer share a sleeping space with their mother and what factors play a role in ITN allocation at the household level. The underlying assumption in any case is that when an eligible woman attends ANC or a child attends EPI for their vaccination, they are offered an ITN; evidence from ANC net voucher schemes in Ghana and Tanzania suggests that this is not always the case [42-44], although there is less evidence from direct delivery of ITNs through these delivery channels.

## Conclusions

Attendance at ANC and EPI are not independent, and therefore the majority of women will have the opportunity to receive ITNs through both services. Although attendance is not independent, delivery through both systems may still add incrementally to delivery through one alone. Therefore, there is potential to increase the proportion of women and children receiving ITNs by delivering through both of these channels. However, modelling is required to determine the level of attendance and incremental potential at which it is cost effective to deliver through both ANC and EPI.

## Competing interests

The authors declare that they have no competing interests.

## Authors' contributions

MC designed and carried out the statistical analysis and drafted the manuscript with assistance from LSP. JB and JW participated in the statistical analysis methods. JL conceived the study and participated in the statistical analysis methods. All authors read and approved the final manuscript.
